# Alpine glacier-fed turbid lakes are discontinuous cold polymictic rather than dimictic

**DOI:** 10.1080/20442041.2017.1294346

**Published:** 2017-05-02

**Authors:** Hannes Peter, Ruben Sommaruga

**Affiliations:** ^a^ Institute of Ecology, Lake and Glacier Ecology Research Group, University of Innsbruck, Innsbruck, Austria; ^b^ Stream Biofilm and Ecosystem Research Laboratory, Faculty of Architecture, Civil and Environmental Engineering, École Polytechnique Fédérale de Lausanne, Lausanne, Switzerland

**Keywords:** Alpine lakes, climate change, glacier retreat, mineral particles, optical properties, thermal structure

## Abstract

Glacier retreat as a consequence of climate change influences freshwater ecosystems in manifold ways, yet the physical and chemical bases of these effects are poorly studied. Here, we characterize how water temperature differs between alpine lakes with and without direct glacier influence on seasonal and diurnal timescales. Using high temporal resolution monitoring of temperature in 4 lakes located in a catchment influenced by glacier retreat, we reported unexpectedly high surface temperatures, even in proglacial lakes located 2600 m a.s.l. Cold glacier meltwater and low nighttime air temperatures caused a distinct diurnal pattern of water temperature in the water column of glacier-influenced lakes. Precipitation onto glacier surfaces apparently leads to rapid cooling of the glacier-fed lakes and disrupts the thermal stratification with several mixing events during the summer. Taken together, these mechanisms contribute to the unique seasonal and diurnal dynamics of glacier-influenced lakes that contrast with the typical dimictic pattern of clear alpine lakes and represent an example of discontinuous cold polymictic lake type. This work contributes to the basic description of how climate and meteorology affect the physical properties of an increasingly common lake type.

## Introduction

The global retreat of glaciers is a prime example of the consequences of climate change (Vaughan et al. 2013) and has been mechanistically linked to anthropogenic activities (Marzeion et al. 2014). Glaciers will continue to shrink, and low-lying glaciers are likely to completely disappear within the next decades. Vanishing glaciers affect the ecology of glacier-influenced freshwater ecosystems in manifold ways (Sommaruga 2015). In addition to the loss of freshwater storage and alterations to the hydrological cycle (Immerzeel et al. 2010), glacier retreat causes bedrock erosion, liberates ions and pollutants into meltwater (Rogora et al. 2013, Pavlova et al. 2014), and threatens the biodiversity of specialized communities (Jacobsen et al. 2012, Wilhelm et al. 2013, Peter and Sommaruga 2016). Glacier retreat, however, also leads to the creation of numerous new lakes in previously ice-covered terrain (Linsbauer et al. 2012). In fact, most lakes on Earth are of glacial origin (Wetzel 2001a) and have formed as a consequence of the retreat of glaciers at the end of the last glacial period.

One outstanding characteristic of glacier-influenced lakes is the brown/gray to turquoise hue of the water, a consequence of the high loads of mineral suspensoids, so called “glacier or rock flour,” which is formed by rock erosion at the glacier base and transported by proglacial streams and runoff. These highly abundant particles are typically small, ranging in size between clay and fine silt, whereas their mineralogical characteristic depends on the lithography of the local bedrock (Sommaruga and Kandolf 2014). Turbidity by mineral particles reduces photosynthetically active radiation (PAR) and ultraviolet radiation (UVR) in proglacial freshwater ecosystems (Donohue and Garcia Molinos 2009, Rose et al. 2014).

Although we have begun to understand the effects of turbid glacier meltwater on the ecology of recipient lakes (Koenings et al. 1990, Hylander et al. 2011, Sommaruga and Kandolf 2014, Sommaruga 2015, Drewes et al. 2016, Peter and Sommaruga 2016), the physicochemical effects of this kind of input to lakes have received less attention. Despite early recognition that turbidity of river water affects intrusion depth (Forel 1885), resulting in complex patterns of overflow and underflow in glacier-fed lakes (Smith 1978, Irwin and Pickrill 1982, Weirich 1986) and the absence of summer stratification in shallow turbid lakes in the Arctic (Brewer 1958, Livingstone et al. 1958), how the thermal structure of the water column is influenced by the current melting of glaciers in proglacial lakes has not been addressed. This lack of information is also reflected in the absence of proglacial lakes in recent meta-analysis and data-collection efforts (Blenckner et al. 2007, Sharma et al. 2015, Woolway et al. 2016).

Discharge of glacier meltwaters is typically cold, with large longitudinal dynamics driven by topography and highly variable flow in glacier forefields (Magnusson et al. 2012). Given the rapid surface flow over impermeable bedrock or thin alpine soils, temperature variability in proglacial streams also rapidly responds to precipitation events (Brown and Hannah 2007). These dynamics directly affect temperature in both ice-contact and distal glacier-fed lakes, the distance to the glacier being important in the latter case (Smith and Ashley 1985). Comparing 50 lakes in Alaska, Koenings et al. (1990) reported a 1.1 °C lower mean water temperature in turbid than in clear lakes. Temperature and turbidity of glacier meltwaters can vary considerably on seasonal and diurnal timescales, resulting in complex patterns of underflow, interflow and overflow in glacier-fed lakes (Irwin and Pickrill 1982, Chanudet and Filella 2008). More recently, the effects of hydropower operations on turbidity and thermal stratification in lakes connected by pump-storage schemes have been explored (Finger et al. 2006, Bonalumi et al. 2012).

Here, we contrast temperature records of 4 lakes influenced by glacier retreat over the course of the ice-free season. One lake has already lost its hydrological connectivity to the glacier and is transparent, thus reflecting the final stage of a glacier-retreat chronosequence. The other lakes are located along an altitudinal gradient below the glacier and represent a gradient in turbidity. We expected that cold glacier meltwaters decrease water temperature in proglacial lakes and that turbidity increases light attenuation, thereby reducing temperature in deep layers of glacier-influenced lakes. The interplay between surface warming and cooling of lake water by glacier meltwater discharge may represent an atypical form of lake mixing pattern for temperate alpine lakes, which are characterized by a weak thermocline (Catalan et al. 2002).

## Study site

**Figure 1.**
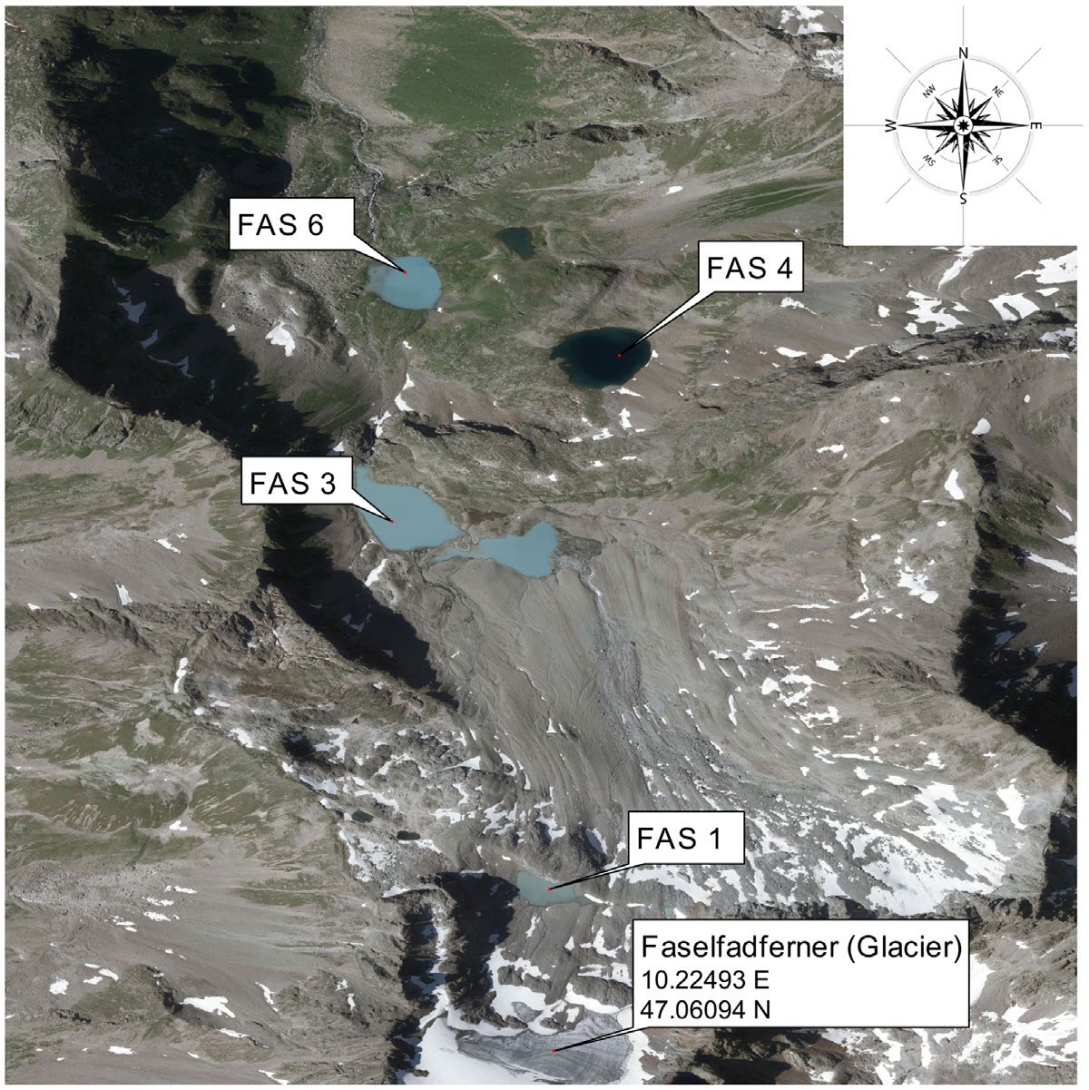
Orthophoto showing the Faselfad catchment comprising the 3 turbid lakes, FAS 1 (2600 m a.s.l.), FAS 3 (2400 m a.s.l), and FAS 6 (2200 m a.s.l.), and the clear lake, FAS 4 (2400 m a.s.l.). FAS 1 and FAS 3 are connected by a stream that, depending on time of year, flows partially below surface. FAS 4 and FAS 6 are also connected by a partially subsurface stream. Orthophoto source: http://www.tirol.gv.at/tiris.

We monitored temperature in 4 alpine lakes (i.e., located above treeline) influenced by glacier retreat. The study area comprises the Faselfad lakes (FAS; Fig. 1), a group of 6 lakes situated between 2263 and 2620 m a.s.l in the western Austrian Alps (47°4′N, 10°13′E). All lakes originated from a rapidly retreating glacier, the Faselfadferner (Drewes et al. 2016), a small glacier located on a steep slope. The Faselfad lakes differ in connectivity to the glacier. Thus, FAS 1 (4 m deep) is the youngest (~40–50 years old) and most turbid lake, located directly beneath the glacier terminus (i.e., proglacial). FAS 2 and FAS 3 (2400 m a.s.l) are located 200 m below FAS 1 and are also fed by glacial meltwater. Depending on water level, these 2 turbid lakes are at certain times connected, and we selected the larger and deeper basin lake, FAS 3 (16 m). Two clear lakes, FAS 4 and FAS 5 (2400 m a.s.l.), are mainly fed by seepage. They became clear after the glacier receded below 2 different rock outcrops, which, judged from the position of moraines, last occurred at the end of the Little Ice Age ca. 1850 (Dr. Jerzy Zasadni, University of Science and Technology in Kraków, pers. comm.). FAS 4 is deep (15 m), but FAS 5 is shallow (~2 m) and potentially freezes completely during winter; therefore, we monitored water temperature only in FAS 4. In the lowest lake, FAS 6 (2260 m a.s.l., maximum depth 10 m), water mainly from FAS 3, but also from FAS 4 and FAS 5, is pooled, resulting in intermediate turbidity values. All lakes have low conductivity (<55 μS cm^−1^) and show no significant gradients in the water column (<3 μS cm^−1^).

## Methods

### Lake water temperature

Lake water temperatures were monitored at 15-minute intervals during the ice-free season in 2012 (18 Jul to 2 Oct) using TidbiT 2 loggers (HOBO, Onset Computer Corporation, Bourne, MA, USA) with ±0.2 °C accuracy, moored to a buoy above the deepest point of each lake. Six to 7 sensors were distributed along the water column of each lake (Supplemental Table S1). In the deep lakes FAS 3 and FAS 4, thermistors were installed at the surface, 1 m, 2 m, 8 m, 12 m, and close to the bottom (16 and 15 m in each lake, respectively). Turbid FAS 3 had one additional thermistor fixed at 4 m depth. In shallow proglacial FAS 1, the sensors were installed at the surface and at 1, 1.5, 2.5, 3, and 4 m depth. In FAS 6, temperature was monitored at the surface and at 1, 2, 6, 8, and 10 m depth. The surface thermistors were deployed ~15–20 cm from the lake surface.

### Turbidity and auxiliary data

We conducted 4 field campaigns during the ice-free seasons using helicopter flights. During these expeditions, nephelometric turbidity (NTU) was characterized across the water column using a portable instrument (Turb 430 T, WTW, Germany) that measures 90° scattered “white” light (Tungsten lamp) tailored to measure turbidity caused by small particles, following EPA method 180.1. In addition, a turbidity sensor (YSI 6136, Yellow Springs Instruments, OH, USA) installed on a YSI 6600V2-4 probe was deployed at 1 m depth in turbid lake FAS 3 between 17 July and 1 August. The probe monitored turbidity with an accuracy of ±0.3 NTU and temperature with an accuracy of ±0.15 °C at 30 min intervals. The YSI probe includes a self-cleaning turbidity sensor that measures scattered light emitted by an LED in the near infrared detected at an angle of 90°. The probe was fixed to a buoy (MB-100, NexSens Technology, Fairborn, OH, USA) anchored above the deepest point of FAS 3 and equipped with a solar-powered data logger (SDL500).

Downwelling irradiance was measured using a PUV-501B profiler radiometer (Biospherical Instruments, San Diego, CA, USA). Profiles were taken during clear sky conditions from a boat anchored above the deepest point. Meteorological data, including precipitation (mm), air temperature 2 m above ground (°C), relative humidity 2 m above ground (%), air pressure (hPa), wind direction (°), wind speed (m s^−1^), and global irradiance (mV), were obtained from the nearest weather station (linear distance 6.7 km; Station Galzig, ZAMG) located at 2081 m a.s.l. In mountainous areas, weather conditions, including wind speed and direction, irradiance, and precipitation, can be localized and dependent on terrain slopes. Thus, we did not include the meteorological data in our statistical modeling, but we assumed that precipitation measured at the weather station reflects the situation at the Faselfad lakes and limited our analysis to visual representations of these patterns. Conclusions drawn from these data must be regarded with caution, however.

Statistical analyses, modeling of thermocline depth, and figures were prepared using R (R Development Core Team 2008) and the package rLakeAnalyzer (Read et al. 2011). The thermocline depth model is tailored to interpolate between discrete data points typical for thermistor buoy measurements. This interpolation is achieved by adding weighting to adjacent measurements, which improves the depth resolution (Read et al. 2011). The distance between thermistors in our study increased with increasing depth (Supplemental Table S1), which potentially affects the accuracy of the modeled thermocline depth at larger depths. Moreover, comparison of 3 temperature profiles recorded by the PUV radiometer casts with thermistor data revealed only poor accordance between the measurements and indicated that the model did not capture the thermocline well when water temperatures were low in early October (e.g., 5.8 °C; Supplemental Fig. S1. During the other 2 sampling occasions, the profile did not show a clear stratification pattern, but rather a sharp initial drop in the uppermost water layer followed by a continuous decrease in temperature for several meters. This pattern is also reflected in the thermistor measurements. The model seems to identify the lower bound of the sharp initial drop as the thermocline depth.

## Results and discussion

Considering all data, average temperature was significantly lower in the proglacial lake FAS 1 (4.50 ± 1.66 °C) than in the other lakes (FAS 3: 6.93 ± 2.01°C; FAS 4: 8.40 ± 1.72 °C; FAS 6: 8.12 ± 2.03 °C; ANOVA, Tukeys HSD: *p* < 0.01); however, similar maximum temperatures were recorded at the surface of all lakes (Supplemental Table S1). The uppermost proglacial lake FAS 1 reached 16.65 °C on 20 August 2012 at 1530 h, and comparable temperature maxima were reached at the surface of the lower lying turbid lakes FAS 3 (15.34 °C) and FAS 6 (16.01 °C), as well as in the clear lake FAS 4 (15.29 °C) in the afternoon between 20 and 22 August. In 1 m depth, however, temperature maxima were already considerably lower in the proglacial lake FAS 1 (12.69 °C) compared to temperatures the other turbid lakes (FAS 3: 15.28 °C; FAS 6: 15.38 °C) and the clear lake (FAS 4: 14.80 °C). Maximum temperature at the lake bottom did not exceed 9.90 °C in the shallow (4 m deep) proglacial lake FAS 1, 6.66 °C in FAS 3 (16 m deep), 10.81 °C in FAS 6 (10 m deep), and 8.67 °C in the clear lake FAS 4 (15 m deep).

**Figure 2.**
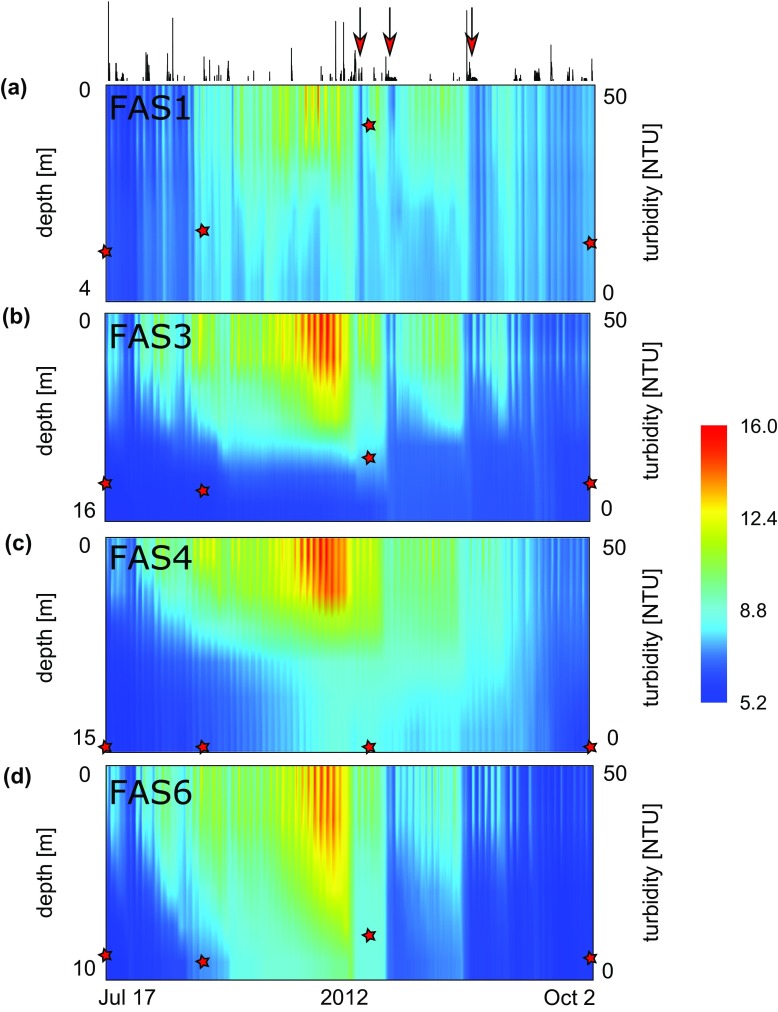
Temperature heat map showing all recorded data of the Faselfad lakes during summer 2012: (a) FAS 1, (b) FAS 3, (c) FAS 4, and (d) FAS 6. The stars overlying the heat maps reflect the trend of mean water column turbidity measured at 4 time points. The line chart on top illustrates precipitation (see Fig. 5 for details). Red arrows highlight 3 occasions of cooling in the turbid lakes, which were absent or less pronounced in the clear lake. Also note the high temperatures at the surface of the glacier-fed lakes and the diurnal patterns at the surface of all lakes. The temperature data are smoothed between depth and over time for visual representation.

Water temperature over the entire ice-free season in the 4 lakes showed a clear temporal pattern, with the highest values found during mid-August (Fig. 2). The peak in water temperature coincided with the maximum turbidity measured in each lake (FAS 1: 49.02 NTU; FAS 3: 17.6 NTU; FAS 6: 12.6 NTU). Throughout the season, temperature at the surface and in 1 m depth in the turbid and clear lake located at the same altitude (i.e., FAS 3 and FAS 4, respectively) were strongly correlated (*R* ^2^_surface_= 0.90 and *R* ^2^_1m_= 0.83, *p* < 0.01). Temperature at the lake bottom of FAS 3 and FAS 4 was more weakly correlated (*R* ^2^ = 0.57, *p* < 0.01), reflecting the seasonal change in the clear lake from 5.8 °C in July to 8.6 °C in mid-August (23 Aug) and 6.1 °C in October. In FAS 3, by contrast, temperature at the lake bottom showed little seasonal variation, varying from 4.2 to 5.8 °C.

All lakes showed a marked diurnal pattern of surface temperature variability (Fig. 2). Comparison of temperature ranges (i.e., maximum–minimum) of the clear (FAS 4) and turbid (FAS 3) lakes revealed that the diurnal ranges in the upper water layers were significantly larger in the turbid than in the clear lake (Table 1). Temperature ranges varied between 1.36 and 2.33 °C in the uppermost 2 m of the turbid lake, whereas temperature varied by 0.6–1.66 °C in the uppermost 2 m of the clear lake. At the lake bottom (i.e., at 16 and 15 m depth, respectively), however, this pattern was reversed. Although the diurnal temperature range was much smaller than at the surface, it was larger in the clear lake (0.38 °C) than in the turbid lake (0.28 °C; Table 1).

**Table 1.** Results of paired *t*-tests between daily temperature ranges (daily max–min) at different depths of turbid lake FAS 3 and clear lake FAS 4. Diurnal changes in temperature were significantly larger in upper layers of the turbid lake than in the clear lake, whereas the range of diurnal temperature variation at the bottom was significantly larger in the clear lake than in the turbid lake. The largest mean difference between the 2 lakes was observed in 1 m depth.

Depth (m)	Median range FAS 3 (°C)	Median range FAS 4 (°C)	Mean difference (°C)	*t*	*p*
0	2.27	1.66	0.61	−11.2	<0.001
1	2.33	1.19	1.15	−15.4	<0.001
2	1.36	0.6	0.76	−15.3	<0.001
8	0.53	0.4	0.14	−3.3	0.002
12	0.47	0.27	0.21	−5.6	<0.001
16/15	0.28	0.38	0.09	3.4	0.001

**Figure 3.**
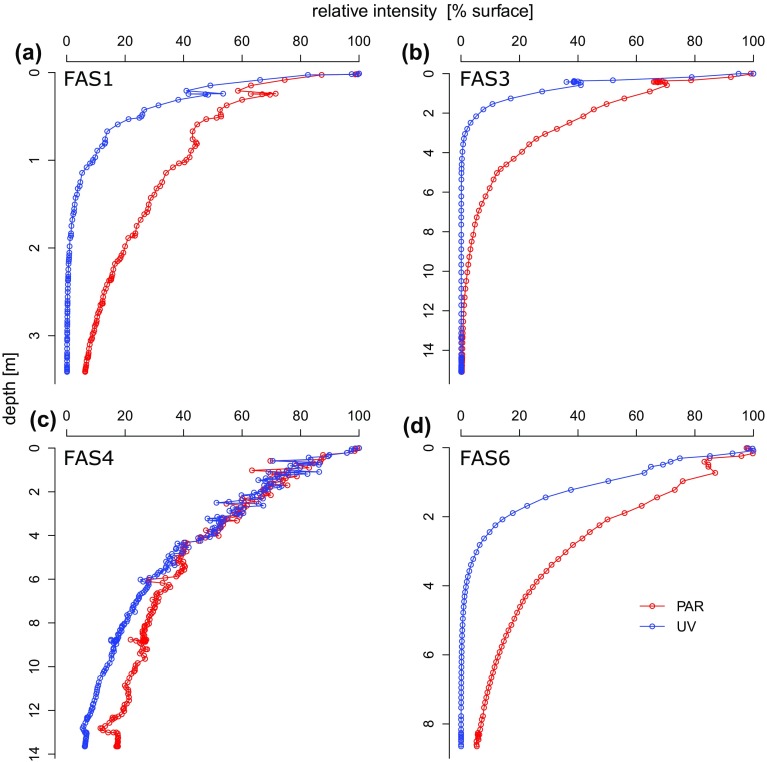
Depth profiles of PAR and UV (nominal wavelength: 320 nm) in (a) FAS 1, (b) FAS 3, (c) FAS 4, and (d) FAS 6 relative to radiation at the surface. Note the rapid attenuation in the turbid lakes FAS 1, FAS 3, and FAS 6, as well as the difference to the clear lake FAS 4.

Heat energy in lakes is driven primarily by solar radiation, diffusive exchange with the atmosphere, and inflowing water. Profiles of PAR and UVR showed a deep penetration in the clear lake, whereas both types of radiation were attenuated rapidly in the turbid lakes (Fig. 3). Solar heating contributes to the warming of deep water layers in wind-protected and transparent alpine lakes (Wetzel 2001b). The rapid attenuation of solar radiation caused mainly by mineral particles seems to reduce the warming of deep water layers in the turbid lake and to exacerbate the heat gain in upper water layers. This observation is plausible considering that both turbid and clear lakes reach similar water temperatures at the surface (Fig. 2). Suspended sediments in the water column (e.g., through sediment resuspension or river discharge) can positively contribute to the heat budget of lakes and coastal oceans, but this is mainly found in systems dominated by organic particles (Donohue and Garcia Molinos 2009).

By contrast, in the Faselfad lakes, rock or glacial flour is characterized by low organic coating (Sommaruga and Kandolf 2014). Nevertheless, turbidity by mineral particles attenuates blue light more rapidly than red light and increases the scattering of sunlight (Donohue and Garcia Molinos 2009). Spectral absorption by mineral particles depends on the elemental composition (mainly the presence of iron), as well as the size and shape of particles (Babin and Stramski 2004). The glacial flour released by the Faselfad glacier is mainly composed of muscovite/illite (57%), chlorite (31%), and quartz (12%; Sommaruga and Kandolf 2014), of which illite contains iron, supporting the notion that suspended particles may contribute to the heating of turbid lake surface layers in this catchment, particularly at times of low meltwater discharge. Density-dependent feedback between water temperature and mineral particle loads may affect particle sinking velocities, however, and thereby also surface temperature in glacier-fed lakes.

We monitored turbidity across the water column during 4 time points but found little vertical variation compared to the large seasonal dynamics (Supplemental Fig. S2). Although we do not have continuous and depth-resolved turbidity data as we do for temperature, we calculated that at the maximum turbidity found (49.02 NTU), which is equivalent to ~0.2 g L^−1^ of glacial particles (Sommaruga and Kandolf 2014), the additional density (assuming a particle density of 2.65 kg L^−1^ and using the formula given in Boehrer and Schultze 2008) will be 0.12 kg m^−3^. This value represents a substantial density change, particularly important to consider when temperatures are ~4 °C and density changes caused by this variable are less pronounced. Automated sampling at higher temporal and vertical resolution of temperature, conductivity, and turbidity in combination with particle trapping will be required to quantify the effects of particle coagulation and sinking, as well as underflow, interflow, and overflow of glacial discharge in the future (Smith 1978, Chanudet and Filella 2007).

**Figure 4.**
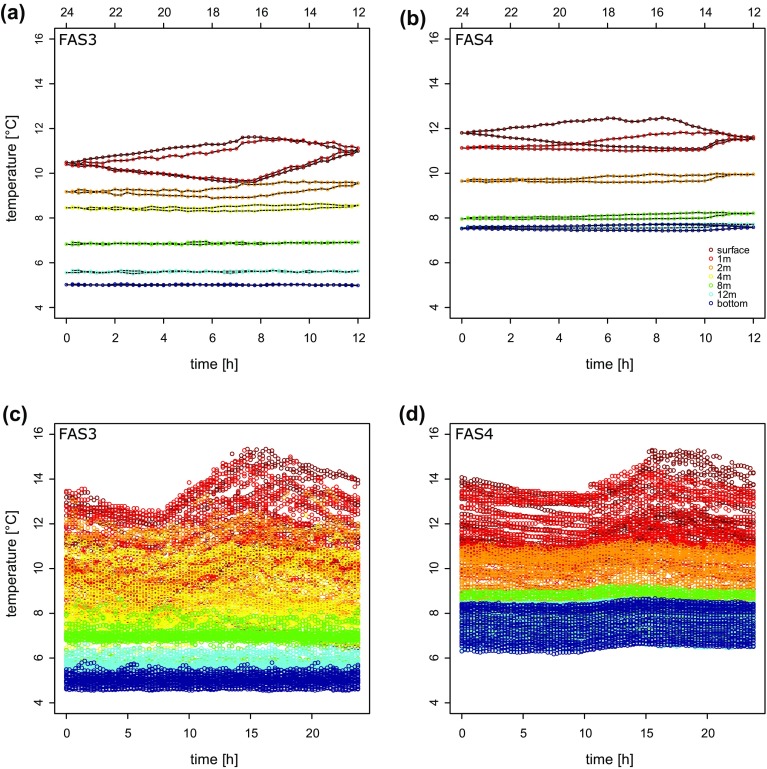
Comparison of average diurnal variation of water temperatures in (a) FAS 3 and (b) FAS 4 along the water column during August 2012. Panel (c) and (d) show all measured data during this period and reflect the variability during this time series. The area of the hysteresis loops (a, b) allow rapid comparison of diurnal variation in the uppermost layers, and the shape of the loop reflects the rates of warming and cooling.

Comparing diurnal patterns of the glacier-influenced (FAS 3) and the unconnected (FAS 4) lakes during peak summer (1–31 Aug) revealed a striking difference (Fig. 4). The maximum rate of change in surface water temperature was higher in the turbid lake (increase: 0.96 °C h^−1^, decrease: −0.57 °C h^−1^) than in the clear lake (increase: 0.64 °C h^−1^, decrease: −0.45 °C h^−1^; Fig. 4). These patterns may be explained by differential shading by the catchment slopes of the 2 lakes, but the temporal dynamics of glacier meltwater discharge may also contribute to these dynamics. For instance, dropping air temperatures may rapidly decrease the amount of meltwater released from a glacier, potentially influencing proglacial lake water temperatures.

In addition to the direct influence of glacier meltwater and turbidity by mineral particles, precipitation onto glacier surfaces may be a driver of downstream temperatures (Brown and Hannah 2007). The potential for a marked cooling of the entire water column is apparent from the temperature heat maps (Fig. 2). During several occasions, pronounced cooling of all turbid lakes can be observed, coinciding with precipitation events. We used meteorological data recorded at a station almost 7 km away and at lower altitude, which limits the explanatory power of this data and the possibility to include wind stress in the assessment. The clear lake showed no such drastic cooling for some of these precipitation events, however, indicating that in addition to other factors such as wind stress, the glacier probably has a pronounced effect on these events.

**Figure 5.**
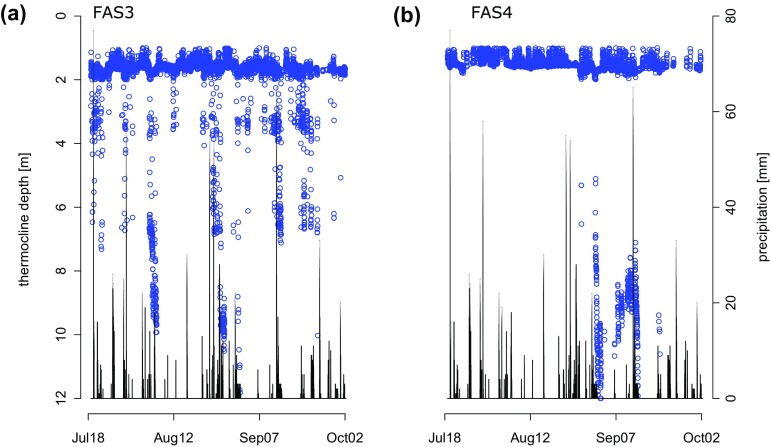
Modeled thermocline depth of (a) turbid lake FAS 3 and (b) clear lake FAS 4 during the ice-free season in 2012 contrasted to precipitation. A deep modeled thermocline is interpreted as a sign of weak thermal structuring. Although the FAS 4 water column is stable for most of the season, it is often disturbed in glacier-fed FAS 3. These events coincide with several precipitation events.

This observation suggests that rain onto glacier surfaces is cooled and thus contributes to the cooling of downstream lakes. To evaluate the magnitude and frequency of cooling events, we modeled the thermocline depth (Fig. 5). In most cases, the modeled thermocline depth of the clear FAS 4 and turbid FAS 3 lakes was at a similar depth (median FAS 3: 1.61 m; FAS 4: 1.48 m; *t*-test *p* > 0.05). The cooling of the water column by precipitation onto the glacier ice may weaken the thermocline, resulting in a greater depth of the modeled thermocline. We interpreted deviations of the modeled thermocline depth (Fig. 5) as a sign for weak thermal stratification, which seems associated with precipitation events throughout the entire ice-free season in turbid lake FAS 3 (Fig. 5). By contrast, in the clear lake, a disruption of the thermal stratification occurred only toward the end of the ice-free season and cannot be associated with precipitation.

Other meteorological factors, such as air temperature, air pressure, and irradiance also affect the thermocline depth over the course of the season (Supplemental Fig. S3). Because weather phenomena can be localized in mountainous areas, however, we cannot tease apart the relative contribution of these factors to the observed variation in thermocline depth or to assess the effects of wind.

**Figure 6.**
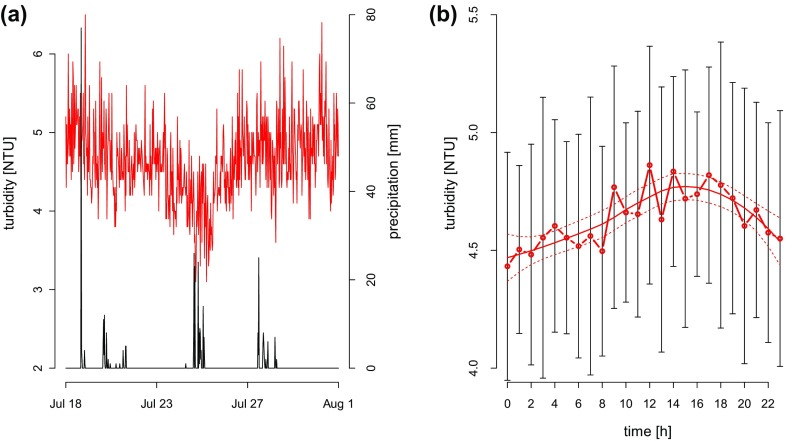
High temporal resolution data of turbidity in 1 m depth of FAS 3 between 18 July and 1 August show dynamic conditions around a mean turbidity of 4.6 NTU (a, red line). A drop to 3.1 NTU on 25 July seems associated with a precipitation event (black line). Despite large variation, turbidity at 1 m depth showed a diurnal trend (b). On average, turbidity (red line with open symbols) was lowest during night and peaked between 1400 and 1800 h. The diurnal trend in mean turbidity is indicated using a local polynomial regression fit (solid red line) with 95% confidence intervals (dotted red line).

To assess the significance, magnitude, and limnological importance of such events, future work should include locally measured meteorological data, ideally conducted at several glacier-influenced catchments. Furthermore, experimental evidence on the effects of mineral particles and their sinking on the density distribution will provide valuable insight into the stability of glacier-fed lakes. In addition, estimates of Schmidt stability (Idso 1973), especially in relation to locally measured wind speed, could further improve our understanding of the temperature dynamics in such types of lakes. The high frequency data obtained with the turbidity sensor placed at 1 m depth in FAS 3 revealed considerable temporal variation (range 3.1–6.3 NTU; Fig. 6a); however, turbidity showed a diurnal trend during this period (Fig. 6b) and was significantly and negatively related with water temperature at 1 m depth (*R* ^2^ = 0.35, *p* < 0.01. Diurnal patterns of turbidity in the center of the lake were complex, potentially driven by many factors we did not account for, including processes of erosion in the glacier forefield and proglacial stream bed channel stability (Nicholas and Sambrook Smith 1998).

Our work reflects the complexity of dynamics of the melting glacier overlain by direct effects on lake water temperature. In conclusion, our study indicates that glacier-influenced turbid lakes feature complex and so far uncharacterized temporal dynamics on diurnal and seasonal scales. Work on proglacial streams has revealed an unexpectedly high temporal heterogeneity of water temperature (Uehlinger et al. 2003, Brown and Hannah 2007, Magnusson et al. 2012), and proglacial lakes may contribute to this heterogeneity. The thermal dynamics found in the Faselfad lakes are different from those of clear alpine lakes (Catalan et al. 2002), differences we attribute to 3 main mechanisms. First, attenuation of solar radiation by mineral particles seems to be an important driver of seasonal temperature increases at the surface, whereas the deeper water layers receive limited energy. The load of mineral particles into the lakes depends on melting of the glacier, which reached a maximum in mid-August. In contrast to clear Arctic and Antarctic lakes (e.g., Brewer 1958, Livingstone et al. 1958), the presence of glacial flour in the Faselfad lakes seems to lead to a gradient in temperature between surface and deeper water layers and to contribute to thermal stratification. Secondly, the diurnal dynamics of freeze–thaw cycles of the glacier influences lake water temperature directly and indirectly through the associated dynamics of turbidity (Milner and Petts 1994). Finally, we attribute the rapid decrease of lake water temperatures along the turbid lake chain to precipitation onto glacier surfaces throughout the entire season. This precipitation results in a particular mixing pattern that can be characterized as discontinuous cold polymictic (Lewis 1983), which represents a rather unique feature of glacier-fed lakes compared to clear, typically dimictic alpine lakes in the temperate zone (Hutchinson and Löffler 1956, Catalan et al. 2002). Summer precipitation in the Austrian Alps is mainly associated with storms, which can be localized. Large amounts of water rapidly flushed from the glacier into the lakes are likely needed to account for the rapid cooling of the water column of lakes located 400 m below the glacier terminus; however, strong winds during the storms can also contribute to the mixing of the water column.

Further generalization of the temporal and spatial patterns reported for the Faselfad lakes may be complicated by changes along altitudinal gradients (Šporka et al. 2006) or with changing lake size (Woolway et al. 2016) or aspect ratio (length:depth). Hydrologic distance to the glacier and catchment morphometry are also factors to consider when modeling water temperature in glacial turbid lakes (Brown and Walsh 1992). Hence, coupled measurements of glacier meltwater discharge with proglacial stream and lake water temperatures will be needed to further assess these patterns. Parametrization and inclusion of the proposed mechanisms may contribute to the accuracy of lake temperature models in remote glaciated areas (Kettle et al. 2004). Because many low-lying glaciers may soon disappear from mountainous landscapes and climate change-related shifts in the magnitude and frequency of precipitation are predicted (Beniston et al. 1997, Beniston 2005), obtaining detailed temporal and spatial records of basic environmental conditions of glacier-fed lakes is an urgent research task. Temperature is a key environmental factor for many ecosystem properties, and thus knowledge on the initial ecosystem state is necessary to anticipate the ecological effects of vanishing glaciers.

## Funding

This work was supported by the Austrian Science Fund (FWF, Project BACK-ALP [grant number P24442-B25]) to Ruben Sommaruga.

## Supplemental data

Supplemental data for this article can be accessed here: http://dx.doi.org/10.1080/20442041.2017.1294346.

## Supplementary Material

Supplemental Data
