# Supplementary material for: Alpine glacier-fed turbid lakes are discontinuous cold polymictic rather than dimictic
Source: Inland Waters. 2017 May 2;7(1):45–54. doi: 10.1080/20442041.2017.1294346 (PMC5478930; doi:10.1080/20442041.2017.1294346)
Supplement: Supplemental Data [file tinw_a_1294346_sm7367.docx]

**Supplementary Material**

**Table S1.** Overview of lake water temperature measured at different depths in the Faselfad lakes during 3 periods in summer 2012.

| Lake | Period | Depth (m) | Temperature (°C)  average ± SD | Temperature (°C)  range |
| --- | --- | --- | --- | --- |
| FAS1 | 18 Jul–1 Aug | 0 | 2.24 ± 0.94 | 0.38–5.15 |
|  |  | 1 | 2.61 ± 0.88 | 1.0–5.14 |
|  |  | 1.5 | 2.48 ± 0.74 | 1.04–4.43 |
|  |  | 2.5 | 2.71 ± 0.62 | 1.34–4.35 |
|  |  | 3 | 2.7 ± 0.61 | 1.34–4.27 |
|  |  | 4 | 2.67 ± 0.6 | 1.29–4.19 |
|  | 1–31 Aug | 0 | 7.05 ± 2.28 | 1.29–16.65 |
|  |  | 1 | 6.72 ± 1.88 | 2.73–12.69 |
|  |  | 1.5 | 5.56 ± 1.14 | 2.72–10.59 |
|  |  | 2.5 | 4.83 ± 0.65 | 3.12–7.02 |
|  |  | 3 | 4.56 ± 0.59 | 2.42–6.97 |
|  |  | 4 | 4.32 ± 0.48 | 3.09–6.91 |
|  | 1 Sep–2 Oct | 0 | 4.99 ± 1.5 | 2.4–9.34 |
|  |  | 1 | 4.84 ± 1.14 | 2.62–7.88 |
|  |  | 1.5 | 4.36 ± 0.82 | 2.58–6.76 |
|  |  | 2.5 | 4.07 ± 0.52 | 2.61–6.28 |
|  |  | 3 | 3.96 ± 0.43 | 2.69–6 |
|  |  | 4 | 3.89 ± 0.4 | 2.8–5.67 |
| FAS3 | 17 Jun–1 Aug | 0 | 7.3 ± 1.28 | 4.74–10.91 |
|  |  | 1 | 7.41 ± 1.16 | 5.04–10.85 |
|  |  | 2 | 6.6 ± 0.71 | 4.93–8.88 |
|  |  | 4 | 6.01 ± 0.52 | 4.87–7.87 |
|  |  | 8 | 5.16 ± 0.31 | 4.45–6.05 |
|  |  | 12 | 4.65 ± 0.17 | 4.25–5.28 |
|  |  | 16 | 4.47 ± 0.15 | 4.19–4.77 |
|  | 1–31 Aug | 0 | 10.62 ± 1.79 | 6.46–15.34 |
|  |  | 1 | 10.56 ± 1.63 | 6.67–15.28 |
|  |  | 2 | 9.24 ± 1.37 | 6.67–13.46 |
|  |  | 4 | 8.44 ± 1.19 | 6.26–12.39 |
|  |  | 8 | 6.87 ± 0.48 | 5.49–8.62 |
|  |  | 12 | 5.6 ± 0.38 | 4.69–6.64 |
|  |  | 16 | 5.01 ± 0.23 | 4.53–5.87 |
|  | 1 Sep–2 Oct | 0 | 7.12 ± 1.41 | 5.1–11.54 |
|  |  | 1 | 7.29 ± 1.31 | 5.35–11.24 |
|  |  | 2 | 6.71 ± 0.92 | 5.35–9.57 |
|  |  | 4 | 6.26 ± 0.66 | 5.23–8.99 |
|  |  | 8 | 5.66 ± 0.24 | 5.05–6.46 |
|  |  | 12 | 5.54 ± 0.2 | 5.02–6.1 |
|  |  | 16 | 5.51 ± 0.18 | 5.0–5.87 |
| FAS4 | 17 Jun–1 Aug | 0 | 8.61 ± 1.27 | 6.36–11.81 |
|  |  | 1 | 7.85 ± 0.81 | 6.37–10.06 |
|  |  | 2 | 6.95 ± 0.56 | 5.82–8.22 |
|  |  | 8 | 6.31 ± 0.35 | 5.67–7.19 |
|  |  | 12 | 6.02 ± 0.27 | 5.57–6.64 |
|  |  | 15 | 5.96 ± 0.27 | 5.44–6.54 |
|  | 1–31 Aug | 0 | 11.75 ± 1.27 | 8.97–15.29 |
|  |  | 1 | 11.3 ± 1.26 | 8.78–14.8 |
|  |  | 2 | 9.75 ± 0.9 | 7.67–11.27 |
|  |  | 8 | 8.05 ± 0.7 | 6.69–9.26 |
|  |  | 12 | 7.62 ± 0.68 | 6.33–8.67 |
|  |  | 15 | 7.56 ± 0.67 | 6.18–8.67 |
|  | 1 Sep–2 Oct | 0 | 8.7 ± 1.16 | 6.69–11.98 |
|  |  | 1 | 8.62 ± 0.98 | 6.78–10.65 |
|  |  | 2 | 8.41 ± 0.89 | 6.76–9.85 |
|  |  | 8 | 8.09 ± 0.71 | 6.69–9.09 |
|  |  | 12 | 7.67 ± 0.51 | 6.38–8.44 |
|  |  | 15 | 7.53 ± 0.46 | 6.18–8.2 |
| FAS6 | 17 Jun–1 Aug | 0 | 8.23 ± 1.18 | 5.75–11.98 |
|  |  | 1 | 8.13 ± 1.04 | 5.86–10.85 |
|  |  | 2 | 7.49 ± 0.88 | 5.77–9.68 |
|  |  | 6 | 6.87 ± 0.8 | 5.44–8.87 |
|  |  | 8 | 6.26 ± 0.64 | 5.23–7.72 |
|  |  | 10 | 5.88 ± 0.36 | 5.26–7.32 |
|  | 1–31 Aug | 0 | 11.08 ± 1.57 | 7.54–16.01 |
|  |  | 1 | 11.01 ± 1.48 | 7.68–15.38 |
|  |  | 2 | 10.39 ± 1.35 | 7.52–13.79 |
|  |  | 6 | 9.8 ± 1.29 | 7.44–12.73 |
|  |  | 8 | 9.18 ± 1.02 | 7.34–11.81 |
|  |  | 10 | 8.57 ± 0.81 | 6.54–10.81 |
|  | 1 Sep–2 Oct | 0 | 7.25 ± 1.18 | 5.28–10.93 |
|  |  | 1 | 7.26 ± 1.04 | 5.45–10.46 |
|  |  | 2 | 6.85 ± 0.85 | 5.33–9.39 |
|  |  | 6 | 6.51 ± 0.74 | 5.26–8.12 |
|  |  | 8 | 6.37 ± 0.65 | 5.26–7.67 |
|  |  | 10 | 6.31 ± 0.57 | 5.23–7.44 |

**Fig. S1.** Comparison between three temperature profiles recorded during PUV casts (small circles) with temperatures measured with dataloggers (large circles). The modeled depths of the thermocline are indicated as horizontal bars.

**Fig. S2.** Depths profiles of turbidity measured during 4 time points over the ice-free season in the turbid Faselfad lakes (a) FAS 1, (b) FAS 3, and (c) FAS 6. Highest turbidity was measured on 28 August in all lakes, whereas turbidity at the beginning and at the end of the ice-free season had similar values in all lakes. Only FAS 3 on 28 August shows a distinct vertical change in turbidity, with a peak at 2 m depth. A slight decrease in turbidity at 5 m depth in FAS 6 on 28 August may be a sign of interflow of clear water (FAS 4) in this lake. Note the different scales in the panels.

**Fig. S3.** Scatter plot matrix of modeled thermocline depth (m) and various meteorological parameters for (a) FAS 3 and (b) FAS 4. Scatter plots are presented for each pair of parameters (below diagonal), and a linear model (red line) is fit. Pearson correlation coefficients are given above the diagonal, with font size relative to *r*. The histograms show the frequency distribution of the respective parameter. See main text for units of the meteorological data. Note that thermocline depth in both lakes is negatively related to temperature, air pressure, and irradiance; however, only in the turbid lake thermocline depth is positively related to precipitation.


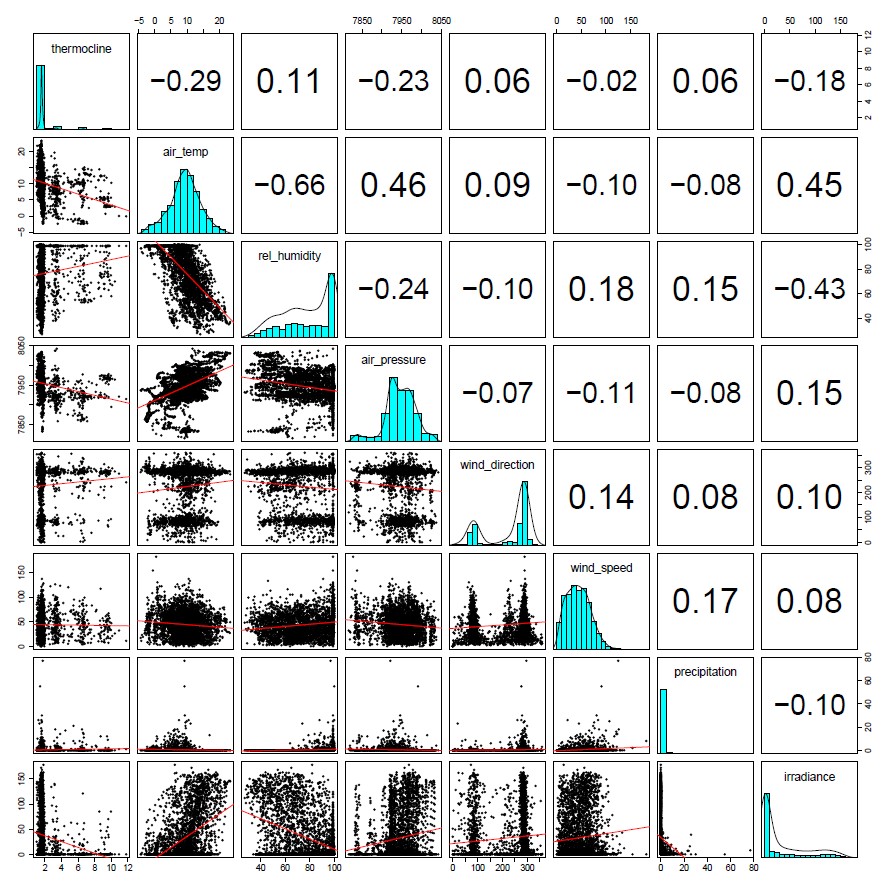


a


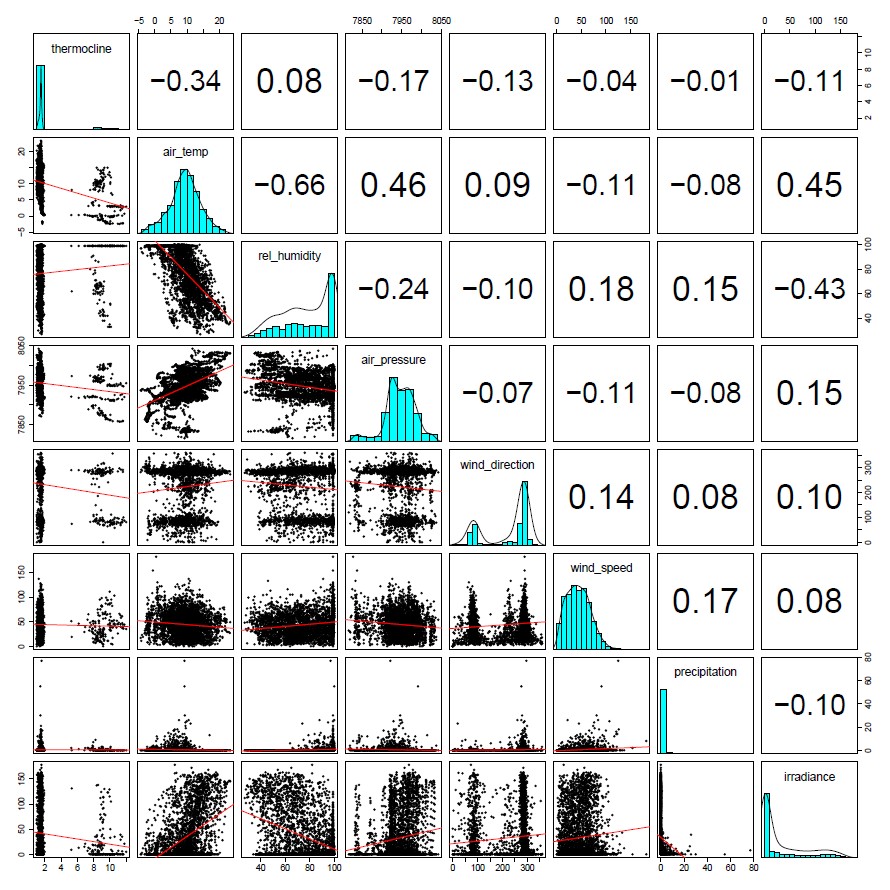


b
